# Road Defect Identification and Location Method Based on an Improved ML-YOLO Algorithm

**DOI:** 10.3390/s24216783

**Published:** 2024-10-22

**Authors:** Tianwen Li, Gongquan Li

**Affiliations:** School of Geosciences, Yangtze University, Wuhan 430074, China; 2023710529@yangtzeu.edu.cn

**Keywords:** improved YOLOv8, spatial attention mechanism, pavement distress detection, target positioning, object detection

## Abstract

The conventional method for detecting road defects relies heavily on manual inspections, which are often inefficient and struggle with precise defect localization. This paper introduces a novel approach for identifying and locating road defects based on an enhanced ML-YOLO algorithm. By refining the YOLOv8 object detection framework, we optimize both the traditional convolutional layers and the spatial pyramid pooling network. Additionally, we incorporate the Convolutional Block Attention to effectively capture channel and spatial features, along with the Selective Kernel Networks that dynamically adapt to feature extraction across varying scales. An optimized target localization algorithm is proposed to achieve high-precision identification and accurate positioning of road defects. Experimental results indicate that the detection accuracy of the improved ML-YOLO algorithm reaches 0.841, with a recall rate of 0.745 and an average precision of 0.817. Compared to the baseline YOLOv8 model, there is an increase in accuracy by 0.13, a rise in recall rate by 0.117, and an enhancement in average precision by 0.116. After the high detection accuracy of road defects was confirmed, generalization experiments were carried out on the improved ML-YOLO model in the public data set. The experimental results showed that compared with the original YOLOv8n, the average precision and recall rate of all types of ML-YOLO increased by 0.075, 0.121, and 0.035 respectively, indicating robust generalization capabilities. When applied to real-time road monitoring scenarios, this algorithm facilitates precise detection and localization of defects while significantly mitigating traffic accident risks and extending roadway service life. A high detection accuracy of road defects was achieved.

## 1. Introduction

With the continuous development of cities, the construction and maintenance of roads has become particularly important. Roads play a vital role in logistics transportation and personnel traffic, and their quality and safety directly affect the production efficiency and safety level of the city. However, due to the complex and changing road environment, frequent vehicle traffic and natural environmental factors, roads are prone to various defects, such as potholes, cracks and settlement. These defects will not only affect the service life of the road, but also may lead to safety accidents. Therefore, timely and accurate identification and positioning of urban road defects is of great significance for ensuring urban safety and extending road service life.

Traditional urban road defect detection methods mainly rely on manual inspection, which is not only time-consuming and labor-intensive, but also subject to the personnel’s subjective judgment, making it difficult to ensure the comprehensiveness and accuracy of detection. With the rapid development of computer vision and machine learning technology, automated road defect detection using intelligent algorithms has become a feasible and efficient solution. W Huang et al. [1] proposed a new method of pavement crack detection using image processing technology. They first preprocessed the images and then connected the cracks using the proposed algorithm. Experiments show that the proposed algorithm is very efficient in highway crack detection. Lin J et al. [2] used nonlinear support vector machine (SVM) to detect potholes. However, since these methods all require manual extraction of highway defect features, which also requires a lot of time and labor costs, most scholars turn to deep learning, which greatly improves the accuracy and efficiency of pavement defect detection by using deep learning networks.

In deep learning networks, a two-stage detection algorithm like Mask R-CNN performs object detection, bounding box regression, and pixel-level semantic segmentation at the same time, which adds additional branch networks, making the number of parameters of the model larger, and higher in computational cost, inference time, and hardware requirements. However, there are also many other low-parameter models. For example, Yang et al. [3] proposed a machine vision-based corrosion and coating defect evaluation method for coal processing plants based on the integration of deep convolutional neural network (cnn) and improved data fusion based on the Dempster-Shafer (D-S) theory. Wang et al. [4], based on the ultrasonic automatic diagnosis method enhanced by continuous wavelet transform and transfer learning deep convolutional neural network, evaluated the compression damage of concrete specimens under temperature changes, and achieved good results, while Wu et al. [5] proposed a lightweight MobileNetV2_DeepLabV3 image segmentation network to extract dam cracks and calculate crack data and achieved good results. YOLO (You Only Look Once) one-stage detection method has been widely used in the field of target detection because of its real-time capabilities, light weight, and high efficiency. Opara J.N. et al. [6] used YOLOv3 to detect damaged asphalt pavement and confirmed that YOLO model has practical application in pavement defect detection. However, the detection accuracy and robustness of the standard YOLO algorithm still need to be improved in the face of complex environments. Fang Wan et al. [7] proposed a lightweight road defect detection algorithm, YOLO-LRDD. By using a new backbone network Shuffle-ECANet, the algorithm reduces the model size while maintaining accuracy and is suitable for deployment on mobile devices. Xu Yi et al. [8] proposed the YOLOv5-PD algorithm for asphalt pavement defect detection, which combined large kernel convolution and channel attention mechanism to improve performance. The average accuracy of the model reaches 73.3% and the inference speed is 41FPS. Wang H. et al. [9] proposed the YOLOv8-D-CBAM algorithm for pavement crack detection, which combined the depth-separable volume product with the convolved block attention module (CBAM) [10] to improve the recognition rate of pavement cracks, achieving a high accuracy rate and a recall rate of more than 98%. The recognition rate of each crack category is above 90%. Previous studies have shown that road defect detection based on YOLO improvement is feasible and efficient. Nevertheless, there remains significant potential for enhancing the accuracy of road defect detection, and research on spatial positioning targeting based on road defect detection is relatively scarce.

Therefore, this paper proposes a road defect location method based on improved YOLO. The specific contributions are as follows:

(1) this study explores a network model based on YOLOv8 and uses SPD-Conv to replace traditional convolution in the backbone, so that it can better capture the details and features of the target, thereby improving the performance of small target detection.
(2) optimizing the space pyramid pool layer in YOLOv8 to effectively reduce the computational complexity and processing time.
(3) Convolutional Block Attention Module (CBAM), which enhances the capture of channels and spatial features, and Selective Kernel Networks (SK) [11], which dynamically adaptively captures features at different scales, are added to the network. The model can not only capture the global information of each channel at different scales, but also pay more attention to the identified target, accurately identify the road defect area, and improve the detection accuracy and robustness of the algorithm in the complex road environment.
(4) combined with the DEM data established by low-altitude remote sensing images of unmanned aerial vehicles, the pixel coordinates of detected road defect images are converted to geographical coordinates based on the location positioning algorithm to realize the spatial positioning of urban road defects, which is conducive to the rapid response and repair of road maintenance departments. Finally, a lot of experiments verify the superior performance of the improved ML-YOLO algorithm in road defect identification and location.

## 2. Materials and Methods

### 2.1. Improved ML-YOLO

**Figure 1:**
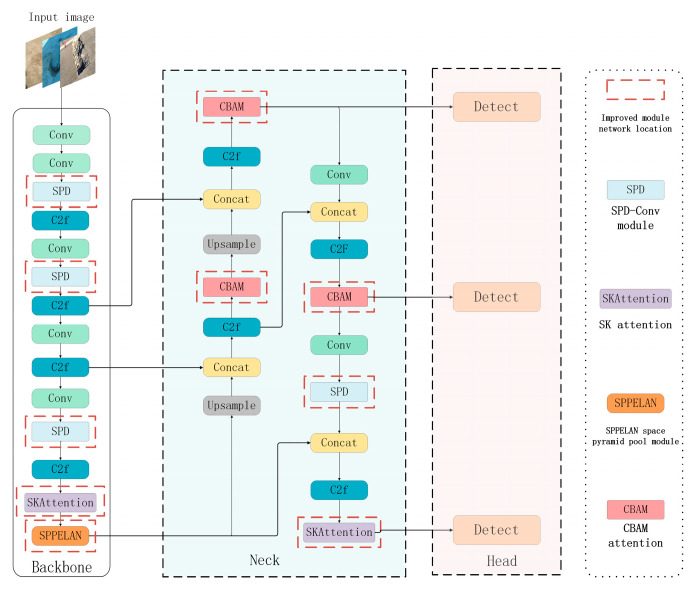
Improved ML-YOLO.

Although YOLOv8 has made many improvements compared with the original YOLO series [12,13,14], road defect identification and location require information extraction in more dimensions, require the model to pay attention to all details of the image, especially small target detection, and do not excessively increase the complexity of the model. Therefore, SPD-Conv is used in the model to replace the traditional convolution in the backbone. It can better capture the details and characteristics of the target, so as to improve the performance of small-target detection. SPPELAN was used to replace the original SPPF to optimize the spatial pyramid pool layer in YOLOv8, so that the model could improve the detection accuracy while maintaining the advantage of computational efficiency. Moreover, SK and CBAM, which enhance channels, space, and multi-scale feature capture, are added to the YOLOv8 model. Through the redistribution of attention to weights, these two modules can help the network better learn the category features and location information of target objects. Considering the characteristics of the attention module, it should be added to the appropriate position of the YOLOv8 model. The characteristics of road defects often have multi-scale characteristics to ensure that better results can be achieved when detecting targets of different sizes. In this study, SK was added to the skeleton and neck of the YOLO model. Adaptive dynamic convolution kernel is used to extract features from road defect feature layers. When the CBAM module is added to the neck of the model, the CBAM attention module can make use of the spatial features such as terrain and defects to improve the defect recognition effect of the model. The figure below (Figure 1) is an improved ML-YOLO model.

### 2.2. Improved Network Module

#### 2.2.1. SPD-Conv Network

**Figure 2:**
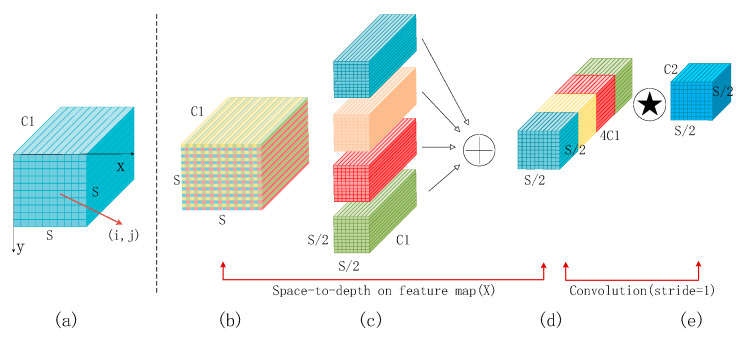
SPD-Conv module structure. (**a**) the original feature map. (**b**) the feature map segmented according to a certain ratio. (**c**) the four sub-feature maps after segmentation. (**d**) the feature map concatenated in the channel dimension. (**e**) the feature map obtained by 1 × 1 convolution.

In convolutional neural networks, cross-row convolution or merge operations are often used to reduce the size of feature graphs, reduce computational complexity, and extract higher-level abstract features. However, this can lead to information loss, which can cause the model to have an incomplete understanding of the input data, which can affect the accuracy of the detection task. To solve this problem, Sunkara R et al. [15] proposed SPD-Conv. Figure 2 shows the structure of the SPD-Conv module. SPD convolution consists of a space-to-depth (SPD) layer and a non-step convolution (Conv) layer. When the SPD layer is applied, the spatial resolution is reduced, but this reduced spatial information is transferred to the channel dimension to be compressed and retained in the deeper channels, so that the number of channels is increased without information loss. A non-step volume layer is applied to this feature graph with an increasing number of channels. This convolution layer does not reduce the spatial size of the feature graph but extracts important features by learning the information in these increased channels. The model can effectively capture the details of small objects at a small spatial resolution [16].

#### 2.2.2. Space Pyramid Pool Improvement

**Figure 3:**
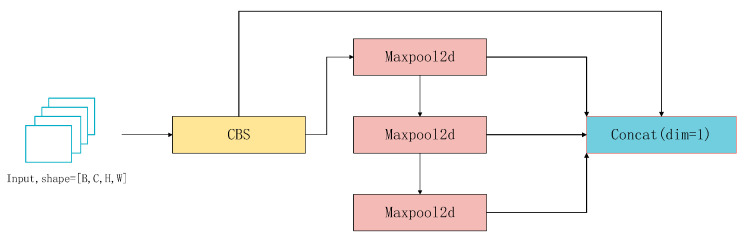
SPPELAN network structure.

In order to improve the recognition accuracy of the model without increasing the number of parameters, in this paper, SPPELAN (Spatial Pyramid Pooling Enhanced with ELAN) replaces the original Spatial Pyramid Pooling Fusion (SPPF) module of YOLOv8. SPPELAN is the latest module introduced in YOLOv9 [17]. SPPELAN is a combination of Spatial Pyramid Pooling (SPP) and Efficient Local Aggregation Network (ELAN). ELAN is a lightweight network structure, which effectively improves the feature extraction capability of the model by means of local aggregation and global integration. By combining SPP with ELAN, the spatial pyramid pooling capability of SPP and the efficient feature aggregation capability of ELAN can be fully utilized to further improve the performance of the model. The introduction of SPPELAN further improves the robustness and generalization ability of the model while maintaining high accuracy. In addition, due to the lightweight nature of ELAN, SPPELAN also helps to reduce the computational effort of the model and increase the speed of reasoning. The network structure of the SPPELAN module is shown in Figure 3.

#### 2.2.3. CBAM Module Introduction

**Figure 4:**
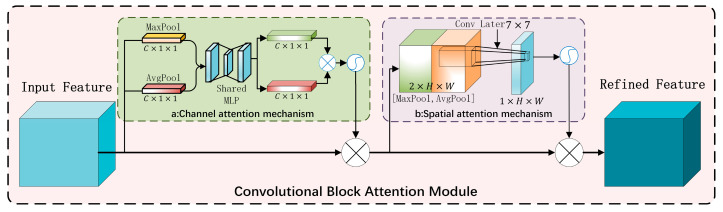
CBAM module network. (**a**) CAM module; (**b**) SAM module.

CBAM (Convolutional Block Attention Module) is an attention mechanism used to enhance the feature representation of convolutional neural networks (CNNS). By calculating the spatial and channel dimensions of the feature graph, we can selectively focus on the important features to improve the performance of the network. Figure 4 is the structure diagram of the CBAM module.

CBAM consists of a channel attention module (CAM) and a spatial attention module (SAM). The main role of the Channel Attention Module (CAM) in CBAM is to enhance the focus on important channel characteristics by evaluating the importance of each channel. Through the global information aggregation of each channel, the CAM module (Figure 4a) can capture the correlation between different channels. Important channels will be given higher weight, which will be more strongly expressed in the feature map, which will help to identify the road defect boundary.

The CAM module implementation idea is as follows:

The input feature graph $F\in R^{H\times W\times C}$ is aggregated by global average pooling ($F_{avg}$) and global maximum pooling ($F_{max}$), and two feature vectors $F_{avg}$ and $F_{max}$ are obtained, respectively

(1) $$F_{avg}=\frac{1}{HW}\overset{H}{\underset{i=1}{\sum }}\overset{w}{\underset{j=1}{\sum }}F\left(i,j,:\right)$$

(2) $$F_{max}=MaxPool\;\left(F\left(i,j,:\right)\right)$$

where i and j are traversal values of the eigenmatrix array. Multi-layer perceptron (MLP) processes $F_{avg}$ and $F_{max}$ through a shared MLP which consists of a hidden layer and an output layer, with the number of nodes in the hidden layer being usually 1/4 of the number of input channels.

(3) $$M_{c}\left(F\right)=\sigma \left(MLP\left(F_{avg}\right)+MLP\left(F_{max}\right)\right)$$

where *σ* stands for the Sigmoid function.

In the CBAM attention mechanism, SAM is a spatial attention module, which makes use of the spatial correlation between road surfaces to enable the model to obtain features for the location of defects in the image, assign attention weights to each spatial location, enhance those spatial locations containing key features, and suppress the feature response of irrelevant or background areas [18]. The model structure diagram is shown in the following figure (Figure 4b).

Similarly, the SAM module also carries out global average pooling and maximum pooling to obtain ${F^{\prime }}_{avg}$ and ${F^{\prime }}_{max}$, and then splicing the pooled feature graphs and performing a 7 × 7 convolution operation. Its main operating formula is as follows:

(4) $$M_{s}\left(F^{\prime }\right)=\sigma \left(Conv\left({F^{\prime }}_{concat},kernel_{size}=7\right)\right)$$

where *σ* represents the Sigmoid function that normalizes the convolutional output to the range [0, 1].

#### 2.2.4. Introduction to SK Attention

**Figure 5:**
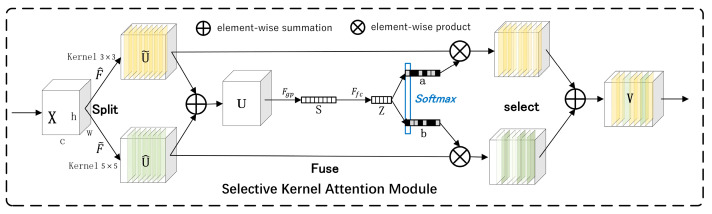
Selective kernel attention module network.

The SK Attention module enhanced the multi-scale feature learning ability of the model by extracting features on multiple convolution cores and dynamically selecting the most suitable convolution kernel size, which significantly improved the model’s ability to extract multi-scale features from road defect areas. The model structure diagram is shown in the following figure (Figure 5).

SK Attention performs convolution operations on input feature graphs through convolution kernels of different sizes to obtain feature graphs $F_{k_{i}}$ of different scales, where $k_{i}$ is the i th convolution kernel. Feature maps extracted from different convolution kernels are fused to obtain F_fuse by adding them one channel at a time. The weight W is obtained by input to the full connection layer after global average pooling of the fused feature maps:

(5) $$W=\sigma \left(FC\left(G\right)\right)$$

The generated selection weights *W* are used to weight the feature plots at each scale to obtain the selected feature plots.

(6) $$F_{\mathrm{selected}}=WF_{k_{i=j}}+\left(1-W\right)F_{k_{i\neq j}}$$

### 2.3. Road Defect Identification Target Location Method

**Figure 6:**
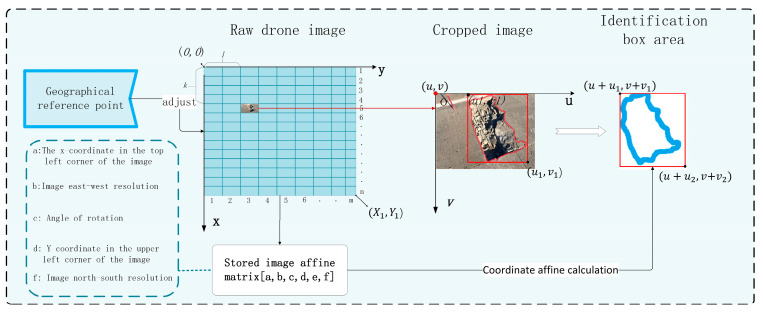
Road defect identification results location method diagram.

Since the image needs to be clipped to the appropriate size when entering the model, the image coordinate information is lost, which affects the geographical location of road defects. In order to solve this problem, inspired by the mine landslide positioning proposed by Lian X et al. [19], this paper proposes an optimized deep learning method to obtain the geographical location of road defects. In order to accurately locate the location of road defects, known geographical reference points and pixel coordinate systems are first used for calibration to ensure the accuracy of the original image projection coordinates. The pixel coordinates are established on the original image, and then the acquired image is cut through image segmentation technology, and the coordinate affine matrix information of the original image is stored. After the clipped image is divided into n rows and m columns, the row information of the image is recorded, namely the number of columns l and the number of rows k, and the pixel coordinate system is established on the clipped image, as shown in the following figure (Figure 6). Using the pixel coordinate data on the original image, the pixel coordinates in the pixel coordinate system of the cropping image are calculated.

(7) $$\left\{\begin{array}{c} U=\frac{lX_{1}}{m}\\ V=\frac{kY_{1}}{n} \end{array}\right.$$

The pixel coordinates $\;(u_{1},v_{1})$,$\;\left(u_{2},v_{2}\right)$ of the left vertex and the bottom point of the recognition frame in the pixel coordinate system of the cropped image are obtained by inputting the cropped image into the ML-YOLO model. Therefore, the pixel coordinates $\;(x_{2},y_{2})$, $\;(x_{3},y_{3})$ of the recognition frame in the pixel coordinate system of the original image can be calculated. Take the left vertex of the identification box as an example:

(8) $$\left\{\begin{array}{c} x_{2}=u+u_{1}\\ y_{2}=v+v_{1} \end{array}\right.$$

The obtained recognition frame coordinates in the pixel coordinate system of the original image. By calculating the coordinates with the stored coordinate affine matrix, accurate projection coordinates can be obtained. Finally, the projection coordinates are converted into geographical coordinates and displayed in the recognition image to obtain the accurate positioning of the recognition results.

(9) $$\left\{\begin{array}{c} X=a+bx+cy\\ Y=d+ex+fy \end{array}\right.$$

where X is the transverse coordinate of the recognition result projection, Y is the longitudinal coordinate of the recognition result projection, x and y are the pixel coordinates of the recognition result in the pixel coordinate system of the original image, and a, b, c, d, e and f are the image affine matrix parameters.

## 3. Experiments and Analysis

### 3.1. Data Set Construction

**Figure 7:**
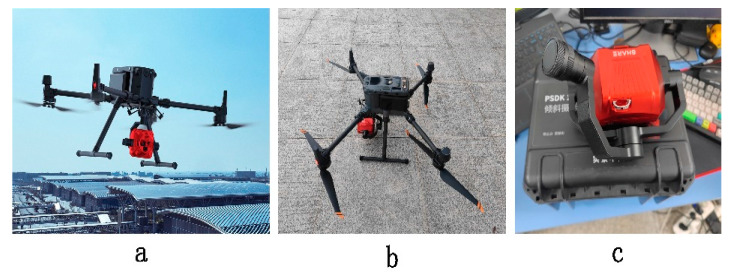
Introduction of the data acquisition equipment. (**a**) DJI official publicity map; (**b**) Real photos by M350 RTK were used; (**c**) Five-lens aerial photography module—102S V3.

In this paper, the road defect sample data set was established based on UAV road inspection images. The UAV equipment was M350 RTK (a and b in Figure 7), designed and manufactured by Shenzhen DJI Innovation Technology Co., Ltd. (Shenzhen, China), and the five-lens tilt camera 102S V3 was used, as shown in the following figure (Figure 7c), which is produced by Shenzhen Saer Intelligent Control Technology Co., Ltd. (Shenzhen, China). The specific parameters are shown in Table 1.

**Figure 8:**
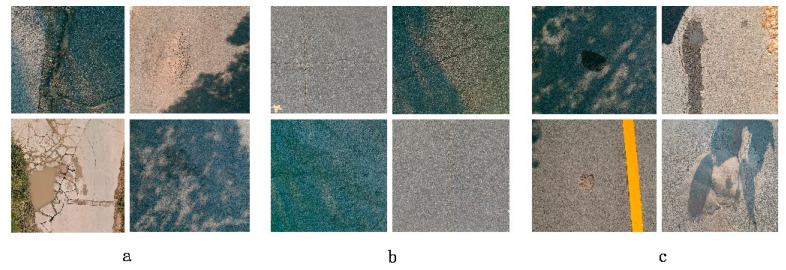
Images of some road defects. (**a**): potholes; (**b**): Cracks; (**c**) bulge.

The image data were obtained from the Wuhan Campus of Yangtze University and its surrounding roads with a spatial resolution of about 0.01 m. The image spatial data was obtained based on Real-time kinematic (RTK) of UAV. Since the original UAV image data is large, it needs to be cropped to a 640 × 640-pixel image, and the color enhancement of the cropped image is carried out at the same time. This paper mainly adjusts the brightness of the image to eliminate the recognition difference of the model under different lighting conditions and adjusts the image contrast to improve the model’s ability to extract different textures and detail features. Part of the original cropped images is shown in Figure 8.

After image preprocessing, this study used LabelMe software (4.5.13) to label the image according to the description of transverse cracks, longitudinal cracks, turtle mesh cracks and potholes according to the specifications of the Highway Technical Condition Assessment (JTG5210-2018) [20], and classified and labeled the road defects into three categories: cracks, potholes, and bumps. The data classification details are shown in Table 2.

### 3.2. Experimental Environment

The experimental environment is shown in the following table (Table 3). The improved YOLO model training was mainly completed under the server of the 64-bit Windows 10 operating system.

The road data set labeled by LabelMe was used in the experiment. The data set consisted of high-resolution UAV aerial images. In order to ensure the generalization ability of the model, the data set was randomly divided into training set (70%), verification set (20%), and test set (10%). Parameter configuration of the experiment is shown in Table 4 below.

### 3.3. Evaluation Index

In order to verify the performance of the model in the task of road defect identification, the commonly used evaluation indexes are selected: accuracy P, recall rate R, and average accuracy $mAP_{@0.5}$. The evaluation index formula is as follows:

(10) $$P=\frac{TP}{TP+FP}$$

(11) $$R=\frac{TP}{TP+FN}$$

(12) $$mAP=\frac{1}{N}\sum_{i=1}^{N}AP_{i}$$

In the formula, *TP* represents the number of positive sample prediction correct, *FN* represents the number of positive sample prediction errors, and *FP* represents the number of negative sample prediction errors.

### 3.4. Results and Analysis of the ML-YOLO Model

#### 3.4.1. Contrast Experiment

In order to further compare the recognition performance of the ML-YOLO model in road defects, the experiment conducted a comparison experiment between the ML-YOLO model, Fast R-CNN, and YOLO series models, and the experimental results are shown in Table 5. The number of parameters of Fast R-CNN is smaller than that of YOLOv8x, but the evaluation results show that the former model is better than the latter. Since ML-YOLO adds a spatial attention mechanism, it has a good recognition effect in various road scenes. When the recognition box overlap threshold is 50%, the average accuracy of target recognition reaches 0.817, the accuracy reaches 0.841, and the recall rate is 0.745. The model effect of YOLOv6n in the road defect recognition scene is poor, as its target recognition accuracy is only 0.691, the average accuracy is only 0.706, and the recall rate is 0.734. ML-YOLO is the best performing of all models, with both accuracy, recall, and mAP significantly higher than other models, indicating superior capability in target detection tasks. The YOLOv8x, as a model with a large number of parameters, also showed better performance, especially in terms of recall and mAP, but failed to surpass ML-YOLO. The Fast R-CNN has some advantages in accuracy, but the overall performance is not as good as the ML-YOLO and YOLOv8x. Although YOLOv6n and YOLOv7n have certain application value in cases of limited resources, their performance is relatively average, especially in terms of recall rate and mAP, and it is difficult to compare it with the ML-YOLO model. Overall, ML-YOLO has a large number of parameters, but it has the most outstanding performance in recognition.

**Figure 9:**
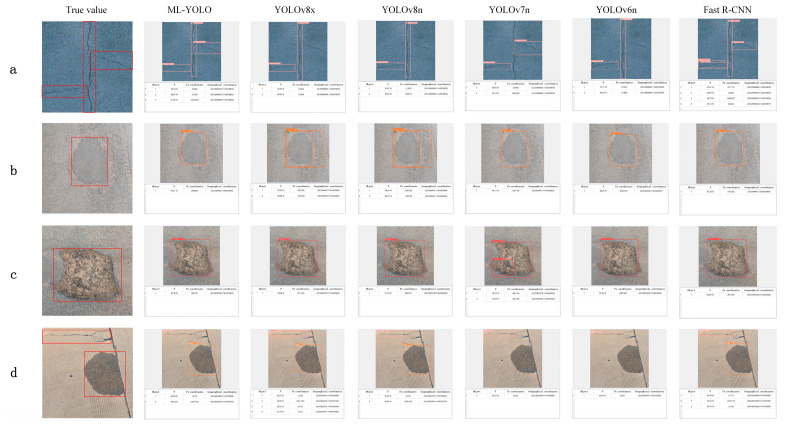
Comparison of road defect identification and location results by different models. (**a**) Cracks; (**b**) Bulge; (**c**) Pothole; (**d**) Multiple types.

The ML-YOLO model and other deep learning models were used to conduct a training pre-test on the road defect data set, and the performance of the ML-YOLO algorithm was visually demonstrated. It is evident that ML-YOLO successfully identified all cracks in the road crack detection task, whereas the unimproved YOLO series models failed to detect all cracks. Among these, only the two-stage detection algorithm Fast R-CNN managed to identify cracks completely, albeit with suboptimal accuracy. In terms of protrusion recognition, ML-YOLO achieved an impressive accuracy rate of 0.88 without any instances of repeated detection frames. When it comes to pothole detection, the enhanced ML-YOLO demonstrated superior accuracy in identifying potholes. Furthermore, in complex multi-type detections, ML-YOLO consistently maintained optimal recognition performance. Part of the visual comparison results are shown in the figure below (Figure 9). Comparing the reference value with the recognition area of the model, it can be seen that the recognition effect of ML-YOLO model is better, and the error recognition and missing recognition are lower. In different terrains and environments, the ML-YOLO model shows high robustness and stability, can accurately display the location of the identification target, and can adapt to a variety of complex road defect identification tasks.

#### 3.4.2. Generalization Experiment

In order to further verify the robustness and detection performance of different data sets of the ML-YOLO model proposed in this paper, the open data set UAPD was used for testing [21], which was taken by UAV in Dongji Avenue, Nanjing, China. The image size of the dataset is 512 × 512, with a total of 2401 pictures, including complex scenes such as different weather, different lighting, trees, and vehicle occlusion [22]. Since the original UAPD data set has more label categories than the ones designed in this paper, it is necessary to unify the label categories, and the final label categories were divided into three categories: crack, pothole, and uplift. Two thousand road defect images were sorted out, and the training set, verification set, and test set were divided in a 7:2:1 ratio. The experimental results are shown in Table 6. It can be seen from the experimental results that there is little difference between the two models in the accuracy and recall rate of crack recognition, and ML-YOLO performs slightly better on mAP@0.5. When identifying potholes, ML-YOLO is significantly better than YOLOv8n on all indexes, especially the accuracy and mAP@0.5, which improved by 0.16 and 0.05, respectively, indicating significant improvement on pothole detection. The accuracy, recall rate, and mAP@0.5 of ML-YOLO in bulge recognition were higher than those of YOLOv8n, especially the recall rate. ML-YOLO is superior to YOLOv8n in all evaluation indexes, which shows the superiority and generalization ability of the improved model in the overall road defect detection.

**Figure 10:**
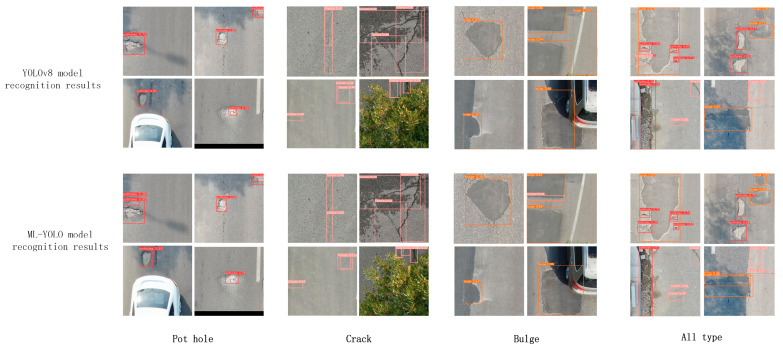
Visualization results of the generalization experiment between ML-YOLO and YOLOv8 model in the UAPD dataset.

To facilitate a more intuitive comparison of the generalization capabilities between the enhanced ML-YOLO and the baseline YOLOv8, particularly regarding their recognition performance across various complex environments, including scenarios involving object occlusion, as well as multi-object and multi-type situations, the following figure (Figure 10) illustrates the defect detection efficacy of different models in classifying cracks, potholes, and bulges under diverse scenes and lighting conditions. As depicted in the figure, ML-YOLO demonstrates a significant recognition advantage over YOLOv8 in pothole detection when obstructed by vehicles. Similarly, during crack detection with tree obstructions, ML-YOLO maintains superior recognition accuracy and exhibits an absolute advantage in recognizing multiple types of road defects. This indicates that the improved ML-YOLO algorithm retains a pronounced defect recognition capability within complex road scenes from public datasets, further substantiating its robust generalization ability.

## 4. Discussion

To investigate the influence of specific network modules on the model’s performance, we conducted an ablation experiment with the following components: SPD-Conv, SPPELAN, CBAM, and SK. The evaluation metrics included accuracy (P), recall rate (R), and mean average precision (mAP@0.5). The specific data are shown in Table 7.

The baseline YOLOv8 model, without any additional modules, achieved an accuracy of 0.711, recall rate of 0.628, and mAP of 0.701. Adding SPD-Conv alone slightly improved accuracy but decreased recall and mAP. The introduction of SPPELAN reduced overall performance, likely due to a reduction in feature parameters.

In contrast, adding CBAM enhanced both accuracy and mAP, while SK improved the recall rate significantly. When combining SPD-Conv and SPPELAN, accuracy reached 0.720, recall was 0.653, and mAP was 0.728, demonstrating improved recall. The combination of CBAM and SPD-Conv further increased accuracy.

The mAP results remained stable across multiple configurations, with notable improvements when both CBAM and SK were included, resulting in an accuracy of 0.769 and mAP of 0.787. Experimenting with SPPELAN, CBAM, and SK yielded an accuracy of 0.779, recall of 0.682, and mAP of 0.766, indicating a synergistic effect of multiple modules on performance.

In the final experiment, combining all modules led to optimal performance, achieving an accuracy of 0.841, a recall rate of 0.745, and a maximum mAP of 0.817, demonstrating the comprehensive enhancement provided by the integrated modules.

This study presents the ML-YOLO algorithm as a robust method for the detection and localization of road defects, focusing primarily on surface-level issues such as cracks and potholes. However, we acknowledge the broader scope of road defect detection that encompasses structural integrity and environmental conditions, which are critical for comprehensive urban maintenance and road safety. Our research predominantly emphasizes the identification of surface anomalies, which may not fully capture the complexities associated with deeper structural problems. Aspects such as subsurface defects, which can lead to significant safety hazards, have not been extensively addressed in our current framework.

## 5. Conclusions

This paper proposes a road defect identification and location method based on an improved ML-YOLO algorithm. By introducing SPD-Conv, optimizing spatial pyramid pooling, and integrating CBAM and SK modules, the capture ability of channel and spatial features is enhanced, as well as feature adaptability at different scales. The YOLOv8 model effectively improves the accuracy and robustness of road defect detection in complex environments. The comparison experiment shows that compared with the baseline YOLOv8 model, the accuracy of the improved ML-YOLO model is increased by 0.13, the recall rate is increased by 0.117, the average accuracy is increased by 0.116. The accuracy is up to 0.841, the recall rate is up to 0.745, and the average accuracy is up to 0.817. Generalization experiments showed that the improved ML-YOLO has high generalization ability in public data sets and can adapt to various complex road defect detection tasks. At the same time, this paper integrates the optimized target identification and positioning algorithm into the ML-YOLO model and combines the DEM data generated by the UAV image to complete the real-time positioning of the model after the target identification, which provides the road maintenance department with rapid response and repair technical support. Future research work will further optimize the lightweight design of the model to meet the needs of real-time processing, while exploring more multi-source data fusion methods to further improve the accuracy and reliability of road defect identification and location. In the future, the improved ML-YOLO model can also be applied to other types of infrastructure such as bridges, tunnels, and footpaths. The researchers can study how the model fits into different materials and structural geometry, potentially providing a broader framework for infrastructure monitoring.

## Figures and Tables

**Table 1:** Data acquisition device parameters.

M350 RTK		102S V3	
Weight (including two batteries)	6.47 kg	Camera size	140 × 140 × 80 mm
Maximum take-off weight	9.2 kg	Camera weight	650 g
RTK position accuracy	1 cm + 1 ppm (level) 1.5 cm + 1 ppm (perpendicularity)	Number of shots	5
Maximum flight time	55 min	Effective pixel	Total pixels ≥ 120 million, Single camera pixel ≥ 24.3 million
GNSS	GPS + GLONASS + BeiDou + Galileo	Picture size	6000 × 4000

**Table 2:** Classification information of road defect dataset.

Defect Category	Image Quantity	Defect Description
Crack	520	A linear break in the surface of a road, usually as a long, thin crack.
Pothole	600	A local depression or hole formed when the road surface material falls off.
Bulge	280	A local bulge or uplift on the road surface due to changes or repairs of underground structures.

**Table 3:** Experimental environment hardware and software configuration.

Parameter	Disposition
CPU	Intel (R) Xeon (R) Gold 6348 2.59 GHz
RAM	256 GB 3200 MHz
GPU	NVIDIA GeForce RTX 3090 24 G
cuDNN	8.9.7
CUDA	12.4
Deep learning framework	Pytorch-2.2.1 + python-3.9

**Table 4:** Experimental parameter setting.

Parameter	Description	Disposition
Epochs	Number of training cycles	250
batch	The number of images per batch	10
imgsz	Enter the size of the image	640
workers	Number of worker threads for data loading	8
lr0	Initial learning rate	0.01
momentum	Learning momentum	0.937
box	Box loss gain	7.5
dfl	Class loss gain	0.5
cls	dfl loss gain	1.2

**Table 5:** Experimental comparison of different models.

Model	#Param	P	R	$mAP_{{@}_{0.5}}$
YOLOv8n	3.1 M	0.711	0.628	0.701
YOLOv8x	68.3 M	0.715	0.726	0.760
YOLOv7n	36.5 M	0.704	0.624	0.683
YOLOv6n	4.7 M	0.691	0.706	0.734
Fast R-CNN	42.5 M	0.757	0.679	0.748
ML-YOLO	139.5 M	0.841	0.745	0.817

**Table 6:** Comparison results of generalization experiments between ML-YOLO and YOLOv8 models on the UAPD dataset.

Categories	ML-YOLO			YOLOv8n		
	P	R	$mAP_{{@}_{0.5}}$	P	R	$mAP_{{@}_{0.5}}$
Crack	0.673	0.576	0.624	0.67	0.581	0.596
Pothole	0.832	0.55	0.561	0.672	0.411	0.511
Bulge	0.721	0.723	0.713	0.689	0.596	0.644
All types	0.752	0.65	0.619	0.677	0.529	0.584

**Table 7:** Comparison of ablation experiments of the models.

Experiment	SPD-Conv	SPPELAN	CBAM	SK	P	R	$mAP_{{@}_{0.5}}$
1					0.711	0.628	0.701
2	√				0.739	0.598	0.684
3		√			0.691	0.583	0.648
4			√		0.741	0.622	0.712
5				√	0.715	0.656	0.733
6	√	√			0.720	0.653	0.728
7	√		√		0.774	0.652	0.722
8	√			√	0.734	0.662	0.737
9		√	√		0.740	0.678	0.742
10		√		√	0.718	0.664	0.730
11			√	√	0.769	0.669	0.787
12	√	√	√		0.748	0.650	0.742
13	√	√		√	0.748	0.661	0.741
14		√	√	√	0.779	0.682	0.766
16	√		√	√	0.823	0.688	0.772
16	√	√	√	√	0.841	0.745	0.817

## Data Availability

This data set corresponds to the internal road data of the school and should not be disclosed due to the requirements of the school.

## Abbreviations

The following abbreviations are used in this manuscript.

ML-YOLO	Monitor Look-You Only Look Once
CBAM	Convolved Block Attention Module
SK	Selective Kernel Networks
UAV	Unmanned aerial vehicle
DEM	Digital Elevation Model
mAP	Mean Average Precision
